# Influence of damming on anuran species richness in riparian areas: A test of the serial discontinuity concept

**DOI:** 10.1002/ece3.3750

**Published:** 2018-01-26

**Authors:** Jacquelyn C. Guzy, Evan A. Eskew, Brian J. Halstead, Steven J. Price

**Affiliations:** ^1^ Department of Biology University of Arkansas Fayetteville AR USA; ^2^ Department of Biology Davidson College Davidson NC USA; ^3^ EcoHealth Alliance New York NY USA; ^4^ Graduate Group in Ecology University of California ‐ Davis Davis CA USA; ^5^ Western Ecological Research Center U.S. Geological Survey Dixon CA USA; ^6^ Department of Forestry and Natural Resources University of Kentucky Lexington KY USA

**Keywords:** amphibian, detection, downstream, floodplain, flow regulation, hierarchical Bayesian analysis, occupancy, urbanization

## Abstract

Almost all large rivers worldwide are fragmented by dams, and their impacts have been modeled using the serial discontinuity concept (SDC), a series of predictions regarding responses of key biotic and abiotic variables. We evaluated the effects of damming on anuran communities along a 245‐km river corridor by conducting repeated, time‐constrained anuran calling surveys at 42 locations along the Broad and Pacolet Rivers in South Carolina, USA. Using a hierarchical Bayesian analysis, we test the biodiversity prediction of the SDC (modified for floodplain rivers) by evaluating anuran occupancy and species diversity relative to dams and degree of urbanized land use. The mean response of the anuran community indicated that occupancy and species richness were maximized when sites were farther downstream from dams. Sites at the farthest distances downstream of dams (47.5 km) had an estimated ~3 more species than those just below dams. Similarly, species‐specific occupancy estimates showed a trend of higher occupancy downstream from dams. Therefore, using empirical estimation within the context of a 245‐km river riparian landscape, our study supports SDC predictions for a meandering river. We demonstrate that with increasing distance downstream from dams, riparian anuran communities have higher species richness. Reduced species richness immediately downstream of dams is likely driven by alterations in flow regime that reduce or eliminate flows which sustain riparian wetlands that serve as anuran breeding habitat. Therefore, to maintain anuran biodiversity, we suggest that flow regulation should be managed to ensure water releases inundate riparian wetlands during amphibian breeding seasons and aseasonal releases, which can displace adults, larvae, and eggs, are avoided. These outcomes could be achieved by emulating pre‐dam seasonal discharge data, mirroring discharge of an undammed tributary within the focal watershed, or by basing real‐time flow releases on current environmental conditions.

## 1 INTRODUCTION

Abiotic and biotic conditions in uninterrupted river systems change predictably along a gradient from headwaters to downstream reaches as channel dimensions and canopy openings increase (i.e., the river continuum concept; Vannote, Minshall, Cummins, Sedell, & Cushing, 1980). In natural rivers, this gradient is gradual (Ward & Stanford, 1983). However, almost all large rivers worldwide are fragmented by dams (Poff, Olden, Merritt, & Pepin, 2007), which disrupt the natural continuum. To describe this phenomenon, Ward and Stanford (1983) proposed the serial discontinuity concept (SDC), which is a series of predictions regarding responses of biotic and abiotic variables to dams. These variables include thermal and flow regimes, water quality, substrate, periphyton, organic matter, and planktonic drift, and their recovery depends on dam size, position along the river, and tributary inputs (Ward & Stanford, 1983).

Specifically, the SDC predicts reduced invertebrate species diversity below impoundments because of the disruption to detrital transport, organic matter inputs, nutrient spiraling, and thermal regimes. The SDC also predicts a gradual increase in biodiversity downstream (Ward & Stanford, 1983), although recovery gradients of biota below dams are rarely examined (Ellis & Jones, 2013). The few studies that have examined recovery gradients indicate reduced species richness downstream of dams. More specifically, because of altered thermal conditions and lower habitat diversity, benthic macroinvertebrate diversity is reduced below dams irrespective of dam location and operation (see review by Ellis & Jones, 2013), and species richness recovers with increasing distance downstream of dams (Ellis & Jones, 2013; Tiemann, Gillette, Wildhaber, & Edds, 2004). Similar patterns occur for freshwater mussels (Randklev et al., 2015; Vaughn & Taylor, 1999), fish (Cumming, 2004; Gehrke, Brown, Schiller, Moffatt, & Bruce, 1995; Gehrke & Harris, 2001), and riparian vegetation (Merritt & Wohl, 2006).

Because riparian zones are not as consistently subjected to flow regulation as rivers, the predictions of the SDC as it pertains to floodplains (e.g., Ward & Stanford, 1995a) have not been well tested (Kingsford, 2000). Headwater riparia are thought to have low invertebrate biodiversity because they are limited by low light, reduced nutrient levels, and a lack of spatiotemporal flow predictability. Flow regulation of headwaters is expected to further decrease biodiversity of riparian zones by reducing detrital transport (Ward & Stanford, 1995a). Mid‐order river riparia are thought to have generally low biodiversity because of the overriding negative influence of bank instability; however, almost no data are available to suggest how river regulation influences biodiversity in mid‐order reaches (Ward & Stanford, 1995a). The highest riparian biodiversity is predicted for meandering, high‐order rivers (i.e., those with lotic, lentic, and semi‐lotic habitats), and river regulation on meandering rivers is expected to be most detrimental to species richness because of floodplain isolation below impoundments, with biotic recovery occurring farther downstream of dams (Ward & Stanford, 1995a).

Floods and lateral connections to rivers are important drivers of ecological processes in riparian zones (i.e., the Flood Pulse Concept; Junk, Bayley, & Sparks, 1989). These processes are disrupted by dams, which capture the flood pulse and subsequently reduce floodplain connectivity (Ward & Stanford, 1995a). Consequences of this disruption may be severe for amphibians because riparian wetlands represent critical habitat for many species (Semlitsch & Bodie, 2003). Thus, alteration of rivers through damming can influence semi‐aquatic amphibian populations. For example, the foothill yellow‐legged frog (*Rana boylii*) is more likely to be absent downstream of large dams than in free‐flowing rivers (Kupferberg et al., 2012), and distance downstream from dams is positively correlated with both occupancy and abundance of several anuran species (Eskew, Price, & Dorcas, 2012). In addition, variability in seasonal flows along regulated rivers has been linked with high mortality of both *R. boylii* and the California red‐legged frog (*R. draytonii*; Kupferberg et al., 2012). Riparian amphibian species distributions can be altered by flow regulation (Wassens & Maher, 2011) because they are sensitive to changes in temperature (Catenazzi & Kupferberg, 2013), unseasonable or strongly variable flows (Kupferberg, Lind, Thill, & Yarnell, 2011; Lind, Welsh, & Wilson, 1996), reduced downstream flows (Bateman, Harner, & Chung‐MacCoubrey, 2008), and breeding habitat loss (Lind, Welsh, & Wilson, 1996). However, the predictions of the SDC have yet to be explicitly examined in relation to riparian amphibian communities which are those occupying habitat adjacent to main river channels.

In this study, we evaluated the effects of damming on amphibian communities along a 245‐km river corridor in South Carolina, USA. Our objectives were to test the predictions of the SDC (modified for floodplain rivers; Ward & Stanford, 1995a) using anuran occupancy and species richness data. More specifically, we compare anuran distributions along the Broad and Pacolet Rivers relative to distance upstream and downstream of dams and also evaluate the effects of urbanized land use surrounding each riparian wetland. While the modified SDC predicts alterations in invertebrate species richness as a result of damming, our focus is on anurans.

## 2 METHODS

### 2.1 Study sites

**Figure 1:**
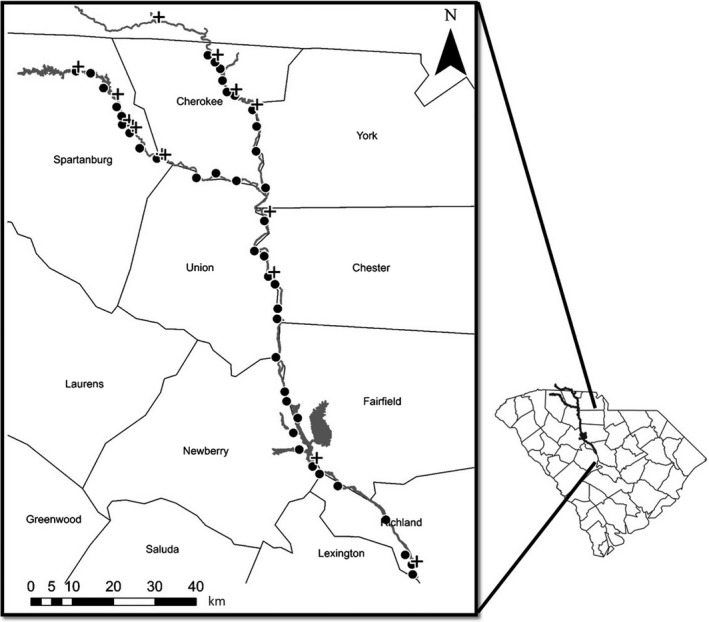
Locations of each anuran study site along both the Pacolet (upper left fork) and Broad Rivers in South Carolina, USA. County boundaries are delineated on the South Carolina outline and are labeled on the inset map. Study sites are shown as black circles, and locations of dams are shown as black crosses. Some of the crosses are obscured because of the proximity of the dams and the scale of the study area. For clarity, the final dam is located downstream of the southernmost site and is not depicted

We used a geographic information system (ArcGIS 10.0; Environmental Systems Research Institute, Redlands, CA, USA), with 30‐m resolution data layers from the National Wetland Inventory (http://www.fws.gov/wetlands/) and the 2006 National Land Cover Database (https://www.mrlc.gov/nlcd2006.php; Fry et al., 2011), to select study wetlands located within the riparian zone of the Broad and Pacolet Rivers, two meandering, high‐order rivers in the Piedmont region of north‐central South Carolina (Figure 1). We define the riparian zone to be any area adjacent to the main river channel or very nearby, but not within the main river bank, with semi‐regular inundation from the river and its tributaries. After locating and ground‐truthing approximately 200 riparian areas as close to the river channel as possible, we eliminated nonaccessible sites and were left with 80 potential study locations. We then generated a circular buffer (1‐km radius) around each site to delineate the distance encompassing the majority of core terrestrial habitat used by most anuran species (Semlitsch & Bodie, 2003). Our final 42 study sites were selected on the basis of spatial independence (i.e., nonoverlapping 1‐km radius circular buffers).

We used aerial photos taken in 2006 to visually identify sixteen dams within our study reach (Figure 1). On the Broad River, we identified nine dams (seven used for hydroelectricity, one as a coal plant cooling reservoir, and one textile mill relic), and on the Pacolet River, we identified seven dams (two for water reservoirs and five originally used in mills). Although the biophysical impacts of a dam vary according to its size and type, we considered the effects of all dams in our analyses because even small dams can influence amphibians (Kirchberg, Cecala, Price, White, & Haskell, 2016), and in preliminary analyses where small dams were removed, our results did not change. Tracing the centerline of the river, we quantified the distance upstream and downstream from each survey site to the nearest dam using the linear referencing tool in ArcGIS. We used the National Land Cover Database (Fry et al., 2011) in ArcGIS to determine percent of urban land cover (i.e., “Developed” land cover classes with low, medium, or high intensity designations) in the 1‐km buffer zone around each site. Use of buffer zones resulted in quantification of urbanization not only in the riparian zone but also in the nearby upland habitats, which are important for anuran species (Semlitsch & Bodie, 2003). In addition, we used a digital elevation model (1:24,000–scale; 30‐m resolution) obtained from the U.S. Geological Survey to calculate differences in mean elevation (within a 50‐m buffer) between each study wetland and the nearest corresponding bank‐full height of the main river channel. Finally, using ArcGIS, we summarized the number of tributaries intersecting the Broad or Pacolet Rivers for each site. More specifically, we quantified the number of tributaries accumulated between each site and its nearest upstream dam. For simplicity, each tributary intersection with the river was counted as “1” regardless of the number of branches feeding the tributary, and we used a Spearman rank correlation in Program R (2.14.0; R Development Core Team, 2015) to establish a relationship between distance downstream of dams and the number of tributaries.

### 2.2 Data collection

We sampled each site nine times using manual calling surveys (Dorcas, Price, Walls, & Barichivich, 2010) to document all species of calling anurans. Surveys lasted for five minutes and were conducted by two experienced anuran surveyors listening independently, recording all species heard, and reconciling any differences before leaving the site. Provided multiple surveys per site and season are conducted, as in our study, surveys of this duration are sufficient for detecting breeding anurans during a given survey occasion (Gooch, Heupel, Price, & Dorcas, 2006). All surveys were conducted between 1845 and 0130 during seasons corresponding to the peak breeding windows for species in our study: spring 2010 (April 13‐May 8), summer 2010 (June 8–24), and winter 2011 (February 21‐March 24). Each site was surveyed three times within each calling window (5–18 days apart), for a total of nine surveys.

### 2.3 Data analysis

We used a hierarchical Bayesian model to estimate anuran species richness and species‐specific occupancy responses to three site‐specific covariates (distance downstream from dam, distance upstream from dam, and percent urbanization) and a survey‐specific covariate (day of year). More specifically, we implemented the species richness model used by Hunt et al. (2013) as modified from Zipkin, Dewan, and Royle (2009). This hierarchical approach treated species‐specific mean occupancy and responses to covariates as originating from an assemblage‐level (i.e., all anuran species together) distribution and thereby estimated both species‐specific and assemblage‐level responses in the same model (Dorazio & Royle, 2005; Zipkin et al., 2009). Our analysis of species richness accounted for imperfect detection of individual species; we therefore did not assume all species were present at every site or that nondetection represented species absence (Dorazio & Royle, 2005). See Hunt et al. (2013) for a detailed description of this model.

We used the following equations to relate species‐specific coefficients (α and β values) to occupancy and detection probabilities (Ψ_*ij*_ and Θ_*ijk*_, respectively) in our model:

$$\text{logit}\;\left(\Psi_{\mathrm{ij}}\right)=u_{i}+\alpha 1_{i}{\text{downdistance}}_{j}+\alpha 2_{i}{\text{updistance}}_{j}+\alpha 3_{i}{\text{percenturban}}_{j}$$

$$\text{logit}\left(\Theta_{\mathrm{ijk}}\right)=v_{i}+\beta 1_{i}\text{cumulativeda}y_{\mathrm{jk}}+\beta 2_{i}{\text{cumulativeday}}_{\mathrm{jk}}^{2},$$

where *i* references species, *j* references sites, *k* references surveys, downdistance was a site's distance downstream from the nearest dam, updistance was a site's distance upstream from the nearest dam, percenturban was the percent of a site's buffer zone containing urban land use, and cumulativeday was defined as days since 1 January 2010. We also included cumulativeday^2^ because anuran species have distinct seasonal calling windows such that a nonlinear trend in detection might be expected (Guzy, Price, & Dorcas, 2014). All covariates were standardized by converting them to *z*‐scores prior to analysis, and data for the Broad and Pacolet Rivers were combined for inference along a 245‐km river corridor.

The model contained the following parameters, specific to each species: *u*_*i*_ *,* α1_*i*_, α2_*i*_, α3_*i*_, *v*_*i*_, β1_*i*_, and β2_*i*_. A final component of the model estimated community summaries (designated with μ), assuming that the species‐specific parameters were random effects, each governed by a community‐level hyper‐parameter. For instance, α1_*i*_ ~ *N* (μ_α1_, σ_α1_), where μ_α1_ is the mean community response (across species) to downdistance and σ_α1_ is the standard deviation in α1 across species (Kéry, Royle, Plattner, & Dorazio, 2009). Because some sites were closer together relative to others, we tested for spatial autocorrelation in our model and found no effect of latitude or longitude on species richness or occupancy and therefore excluded these covariates from our modeling framework.

Our model used uninformative priors for the hyper‐parameters (i.e., U[−5, 5] for α and β, U[0, 10] for σ, and U[−10, 10] for μ parameters), and species‐specific model coefficients were truncated at ±5 from μ to avoid traps. The mean and standard deviation of the model coefficients were calculated, along with the 2.5 and 97.5 percentiles of the posterior distribution, which represent a 95% Bayesian credible interval (CI). We inferred significance for continuous covariates when CIs did not contain zero. Species‐specific occupancy and detection probabilities were derived using the inverse logit transformation. We estimated species richness at sampled sites by summing indicator variables for occupancy for each species at each site and simulated species richness at hypothetical sites from 0.05 to 47.5 km downstream of dams at each model iteration to generate a posterior predictive distribution for species richness as a function of distance downstream of dams.

We organized our data in program R (2.14.0; R Development Core Team, 2015) and executed data analysis in the software program WinBUGS (Lunn, Thomas, Best, & Spiegelhalter, 2000) using R2WinBUGS (Sturtz, Ligges, & Gelman, 2005). The model was run on three independent chains of 300,000 iterations each, after a burn‐in period of 30,000 iterations. Output was thinned by a factor of three, so inference was based upon 300,000 samples from the stationary posterior distribution. Evidence for lack of convergence was assessed by examining history plots and the Gelman and Rubin statistic (Gelman & Rubin, 1992); we found no evidence for lack of convergence (Gelman and Rubin statistic <1.02 for all monitored parameters).

## 3 RESULTS

### 3.1 Site characteristics

Our study sites were 0.05–47.51 km downstream from the nearest dam (mean = 13.47, *SD* = 13.55) and 0.30–50.69 km upstream from the nearest dam (mean = 16.61, *SD* = 14.01). Urban land cover in the 1‐km radius buffer surrounding sites was 0–49.33% (mean = 9.97, *SD* = 12.48). Study sites were 0–550 m from the edge of the river channel (mean = 95.54, *SD* = 127.81) and 2.06–20.47 river‐km from each other (mean = 6.42, *SD* = 4.01). The difference in elevation between our wetlands and the bank‐full height of the river channel ranged from −9.77 to 29.69 m (mean = 10.13, *SD* = 10.19). Among our study sites, the number of tributaries increased farther downstream of dams, and this correlation was highly significant (Spearman's *r*_*s*_ = 0.98, *n* = 42, *p* <0.01).

### 3.2 Anuran detections

We observed 13 anuran species among all sites (Table 1) and, each species’ distribution encompasses our entire study area of north‐central South Carolina, and more broadly, much of the southeastern United States (Powell, Conant, & Collins, 2016). Raw counts of anuran richness per site ranged from two to 12 species. Our median model‐estimated number of species per site ranged from 5 species (95% CI 3 to 8) to 13 species (95% CI 12 to 13). Our model indicated variable occupancy among species, with mean estimated occupancy probabilities ranging from 0.45 (95% CI 0.25 to 0.69) for *Lithobates sphenocephalus* to 0.96 (95% CI 0.86 to 0.99) for *Anaxyrus fowleri* (Table 1). Mean estimated species detection probabilities were also highly variable (Table 1).

**Table 1:** Summary of species observed within riparian zones of the Broad and Pacolet Rivers, South Carolina, USA, and their predominant breeding habitat (Lannoo, 2005)

Species	Occupancy probability			Detection probability			General breeding habitat (Lannoo, 2005)	
	Estimate	Lower 95% CI	Upper 95% CI	Estimate	Lower 95% CI	Upper 95% CI	Predominant Hydroperiod	Preferred Waterbodies may include:
*Acris crepitans*	0.53	0.34	0.73	0.81	0.34	0.96	Permanent or Ephemeral	Lakes, ponds, wetlands, ditches, potholes, floodplains, flooded pastures, canals, river backwaters, sloughs, streams
*Anaxyrus americanus*	0.83	0.56	0.98	0.01	0.00	0.25		
*Anaxyrus fowleri*	0.96	0.86	0.99	0.89	0.61	0.97		
*Anaxyrus terrestris*	0.58	0.15	0.97	0.06	0.00	0.62		
*Gastrophryne carolinensis*	0.83	0.44	0.98	0.43	0.08	0.81	Ephemeral	Meadows, marshes, bottomland swamps, vernal pools, flooded pastures, ditches, sloughs, ponds
*Hyla chrysoscelis*	0.91	0.75	0.99	0.64	0.20	0.90		
*Hyla cinerea*	0.60	0.40	0.78	0.89	0.64	0.96		
*Pseudacris crucifer*	0.86	0.68	0.96	0.01	0.00	0.02		
*Pseudacris feriarum*	0.87	0.72	0.96	0.07	0.00	0.56		
*Lithobates catesbeianus*	0.53	0.36	0.70	0.61	0.15	0.92	Permanent	Lakes, streamsides, permanent wetlands
*Lithobates clamitans*	0.61	0.39	0.82	0.80	0.38	0.94		
*Lithobates palustris*	0.82	0.36	0.99	0.00	0.00	0.20	Permanent or Ephemeral	Ponds, pools, floodplain wetlands, marshes, streamsides
*Lithobates sphenocephalus*	0.45	0.25	0.69	0.04	0.00	0.58	Ephemeral	Shallow, non‐flowing waterbodies

Model‐estimated occupancy and detection probabilities, calculated at mean values of upstream distance from dam, downstream distance from dam, percent urbanization, and cumulative day, are included along with 95% credible intervals for each estimate.

### 3.3 Community‐level summary

When all anurans were considered together, mean response to distance downstream from dam (μ_α1_) was positive with a probability of 0.967 (mean parameter estimate: 0.56; 95% CI −0.02 to 1.27; Table 2), indicating that anurans occurred more frequently farther downstream from dams. Individual species’ responses to the downstream covariate varied somewhat in magnitude as indicated by the across‐species standard deviation (σ_α1_ = 0.79), which was larger than the corresponding mean (μ_α1_) covariate estimate (Table 2). Thus, our model indicated that the mean occupancy response to increasing distance downstream from dams was positive but not consistent in magnitude across species.

**Table 2:** Summary of hyper‐parameters for occupancy (α) and detection (β) covariates for anurans within riparian zones of the Broad and Pacolet Rivers, South Carolina, USA

Community level hyper‐parameter		Mean	*SD*	Lower 95% CI	Upper 95% CI
μ_α1_	Downstream from dam	0.56	0.33	−0.02	1.27
σ_α1_	Downstream from dam	0.79	0.36	0.20	1.63
μ_α2_	Upstream from dam	−0.04	0.18	−0.39	0.31
σ_α2_	Upstream from dam	0.21	0.18	0.01	0.66
μ_α3_	Percent Urban	−1.43	1.23	−3.67	1.09
σ_α3_	Percent Urban	1.34	0.92	0.06	3.43
μ_β1_	Day of Year (linear term)	0.79	0.74	−0.66	2.25
σ_β1_	Day of Year (linear term)	2.25	0.65	1.25	3.79
μ_β2_	Day of Year (squared term)	−1.87	0.98	−3.75	0.14
σ_β2_	Day of Year (squared term)	3.12	0.83	1.90	5.12

The symbol μ indicates a mean community response, while σ indicates the standard deviation in the response to the covariate across species.

The anuran response to μ_α2_, distance upstream from dam, was very close to zero (mean parameter estimate: −0.04; 95% CI −0.39 to 0.31), and the response to μ_α3_, percent urbanization, was negative with a probability of 0.87 (−1.43; 95% CI −3.67 to 1.09; Table 2), suggesting anurans exhibited essentially no response to upstream distance from dams and occurred less frequently at more urbanized locations.

The community response to detection covariates (μ_β1_, cumulative day linear term, and μ_β2_, cumulative day squared term) indicated a weak response (mean parameter estimates: 0.79 [95% CI −0.66 to 2.25] and −1.87 [95% CI −3.75 to 0.14], respectively; Table 2) as both contained positive and negative values in the 95% CI, reflecting uncertainty in the mean community responses. This weak response to cumulative day is not unexpected considering the species we observed have different calling windows (Guzy et al., 2014). Furthermore, there was considerable variation among species’ responses to these detection covariates (Table 2; σ_β1_ = 2.25, σ_β2_ = 3.12).

### 3.4 Occupancy and species richness responses to downstream distance from dam

**Figure 2:**
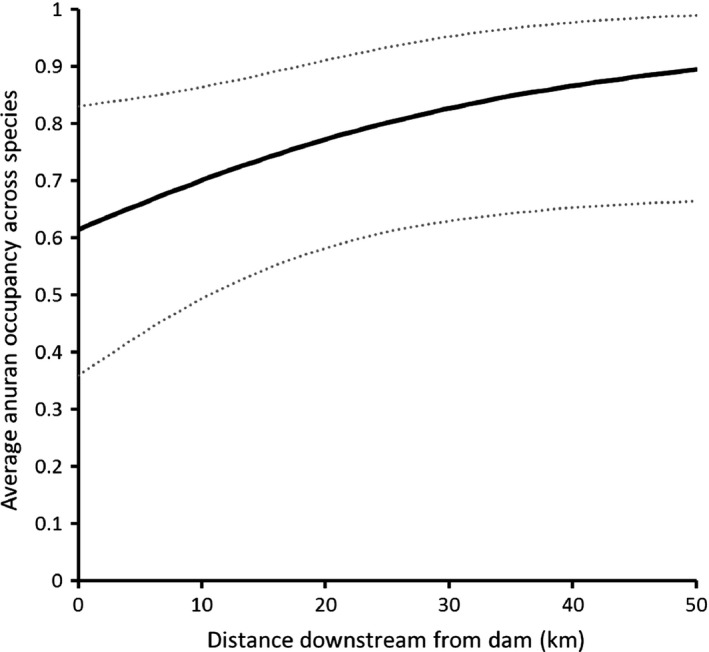
Relationship between mean anuran occupancy probability and distance downstream from a dam in the Broad and Pacolet Rivers, South Carolina, USA. Solid line represents the posterior mean community response, and dashed lines represent a 95% credible interval. Occupancy probabilities were calculated at mean values of upstream distance from dam and percent urbanization

**Figure 3:**
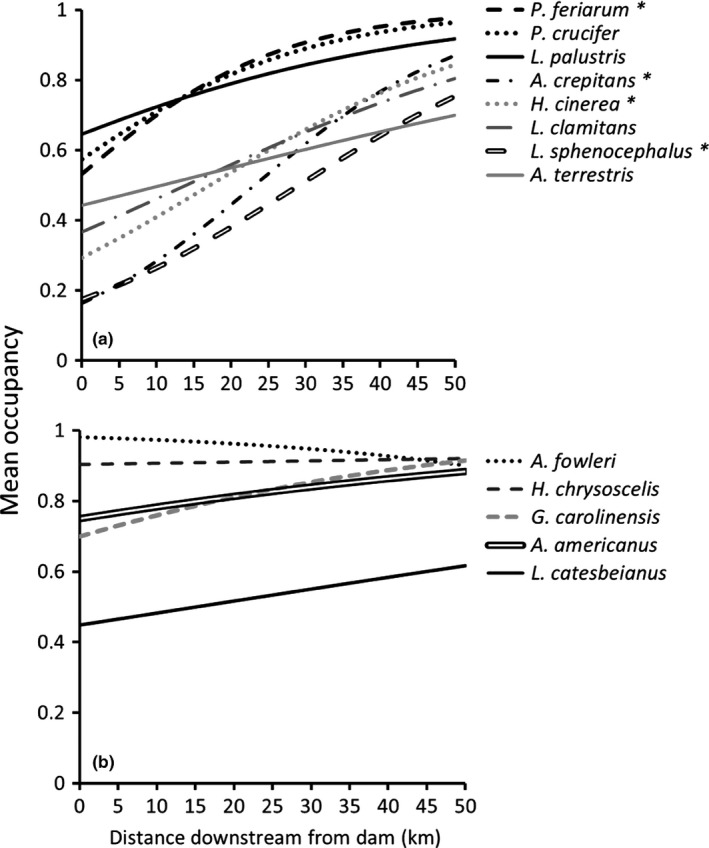
Relationship between mean species‐specific anuran occupancy probability for (a) dam‐sensitive and (b) dam‐insensitive species, and distance downstream from a dam in the Broad and Pacolet Rivers, South Carolina, USA. Occupancy probabilities were calculated at mean values of upstream distance from dam and percent urbanization. Credible intervals are omitted for clarity, and asterisks indicate species for which the downstream distance from dam covariate parameter (α1_*i*_) estimate did not overlap zero

**Figure 4:**
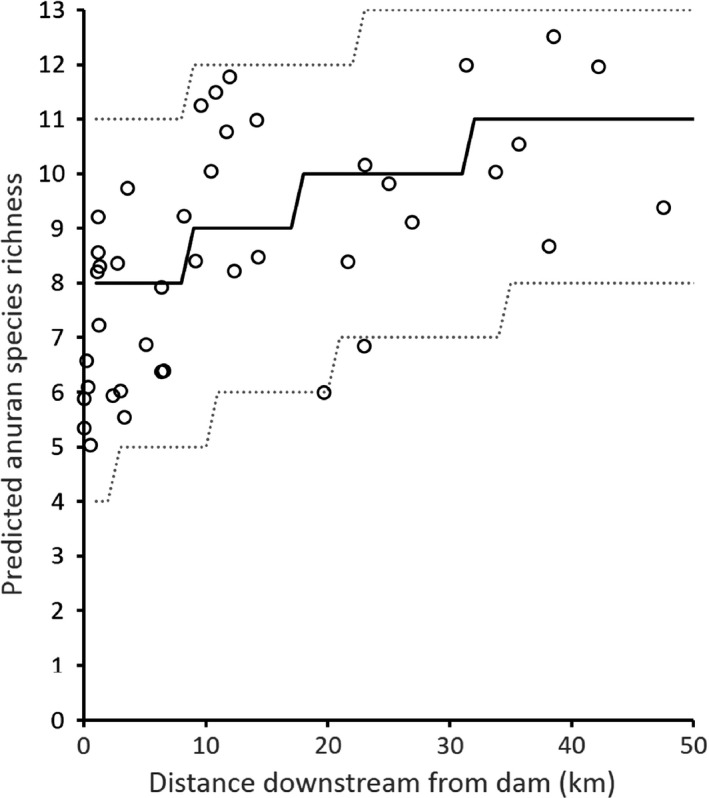
Estimated anuran species richness in riparian zones of the Broad and Pacolet Rivers, South Carolina, USA, in relation to distance downstream from dams. Solid line represents the posterior median, circles are site‐specific mean richness estimates, and the dashed lines represent a 95% predictive interval of species richness at hypothetical sites

We observed a positive mean occupancy response across anuran species to increased distance downstream from nearest dam (Figure 2). Mean estimated occupancy across species increased farther downstream from dams, varying from 0.62 (95% CI 0.36 to 0.83) at a distance of 0.05 km downstream from a dam to 0.90 (95% CI 0.66 to 0.99; Figure 2) at a distance of 47.5 km downstream from a dam. We observed consistent, positive estimates of species‐specific responses to the distance downstream covariate (Figure 3). Similarly, median predicted species richness increased farther downstream from dams, varying from 8 (95% predictive interval 4 to 11) species at a distance of 0.05 km downstream from a dam to 11 (95% predictive interval 8 to 13; Figure 4) species 47.5 km downstream from a dam.

## 4 DISCUSSION

At the spatial extent of our study, which included 42 sites, 16 dams, and approximately 245 km of river, we found a strong downstream effect of damming on riparian anurans, with estimated anuran species richness increasing from 8 species just below impoundments up to 11 species 47.5 km downstream of dams. The threshold length required to achieve maximum species richness was ~40 km. Our results agree with the general predictions of the floodplain‐modified SDC (i.e., species richness is reduced immediately downstream of dams and increases with distance downstream of dams). These findings suggest that river stretches immediately downstream of dams may not provide suitable habitat for some anuran species.

For anurans in our system, the most important consequence of damming is likely its tendency to isolate the river channel from its floodplain. In riparian zones, because of varying water levels, the availability of amphibian breeding habitat is variable from year to year (e.g., Lind et al., 1996). Riparian wetlands are sustained by interactive pathways, including sediment and nutrient deposition occurring during seasonal inundation, when flood pulses form a moving shoreline across the floodplain (Ward & Stanford, 1995b). During these flood pulses, rivers flood their banks, facilitating high levels of aquatic productivity and enhancing connectivity. However, flow regulation by dams reduces connectivity and flood peaks such that river reaches downstream from dams have reduced lateral water flows (Kingsford, 2000; Ward & Stanford, 1995b), which may result in a reduction in area or elimination of riparian‐zone wetlands that provide critical breeding habitat for anurans. During years when flow is lower than average, as in our study, the disconnection of the floodplain from the river is further exacerbated. For example, one study found that toad abundance along a regulated river was low except during the year a flood pulse was released from a local dam, reconnecting riparian‐zone breeding habitats (Bateman et al., 2008). These water releases are beneficial when timed to occur during anuran breeding seasons and, importantly, provide the greatest benefit to anurans when they mimic natural patterns of daily, seasonal, and annual variation in river flow (Kupferberg et al., 2012).

The greatest reductions in connectivity by river regulation are expected to occur in meandering rivers, such as the Broad River, where a multitude of dynamic interactive pathways link the river channel to the riparian habitat (Ward & Stanford, 1995b). Because there are no undammed mid‐ to high‐order rivers in the Piedmont region of the USA, data on reference conditions (i.e., anuran species richness of undammed rivers) are unavailable; thus, we cannot provide information on anuran recovery gradients in our study system. However, we do provide evidence for a strong downstream damming effect, with species richness peaking 47.5 km downstream of dams. At this downstream distance, tributaries and lateral connections to the floodplain may begin to accumulate, restoring flow and sediment transport (Ward & Stanford, 1995b) such that the riparian habitats become more diverse (i.e., extensive vegetation along the river's edge, isolated pools, and ephemeral wetlands). Although not measured in our study, we suggest that increases in habitat heterogeneity facilitate increases in anuran richness downstream of dams. For example, floodplains facilitate the creation and maintenance of a variety of waterbodies with varying degrees of connectivity to the main river channel (Ward & Stanford, 1995b) that are favorable for amphibians (Indermaur, Schmidt, Tockner, & Schaub, 2010). This diversity of waterbodies is particularly important for anurans because they vary in their breeding habitat requirements and are influenced by wetland depth, vegetation structure, canopy cover, and amount of woody debris (e.g., Grant, Otis, & Koford, 2015). Perhaps most importantly, some species breed in wetlands while others utilize the riparian edge of the river channel (Peterman, Anderson, Drake, Ousterhout, & Semlitsch, 2014). Such a degree of habitat variability generally does not occur immediately downstream of dams.

Increases in river‐floodplain connectivity can be driven by an increase in the number of tributaries farther downstream of dams, which reset ecological conditions toward natural or unregulated conditions (Stanford & Ward, 2001). Among our study sites, the number of tributaries increased farther downstream of dams. Tributaries support important ecological functions (e.g., they supply water, sediment, and organic matter) and provide unique habitats to support amphibians (Rice, Kiffney, Greene, & Pess, 2008). For example, tributaries may be exploited by mobile species (Power & Dietrich, 2002), such as *R. boylii*, a species that spends much of its time in tributary streams but uses the river‐tributary confluence and main stem rivers primarily for breeding (Kupferberg, 1996).

Additionally, riparian anuran communities immediately downstream of dams can be negatively influenced by disruption of the predictable annual flood‐drought cycles with which they evolved (Bunn & Arthington, 2002; Lytle & Poff, 2004). Hydrologic alteration was associated with decreases in the distribution and abundance of *R. boylii* and *R. draytonii*, likely in response to disruption of the seasonal synchrony between stable low‐flow conditions and reproduction (Kupferberg et al., 2012). Reproduction in many taxonomic groups is timed to avoid flow fluctuations in rivers with seasonally predictable flooding. However, immediately downstream of dams, the potential for anurans to adjust reproductive behaviors may be constrained by a lack of environmental cues. Seasonal cues (e.g., day length, temperature) that trigger migration, and in‐stream cues (e.g., stream depth, velocity) that influence oviposition site selection (Grabowski & Isely, 2007; Kupferberg, 1996) can become decoupled from the conditions offspring may experience, with the result that there may be no indication of a water release or drawdown prior to its occurrence. For example, if a threshold temperature or water level is required before frogs can initiate breeding and these conditions occur just prior to a high‐flow release, egg masses or larvae are likely to be lost (Lind et al., 1996).

In a concurrent study of the same 13 species examined here, Eskew et al. (2012) found that occupancy of two anuran species (*Acris crepitans* and *Lithobates sphenocephalus*) increased with increasing distance downstream of dams, and a similar pattern was observed for abundance of six species. Our main objective was to test the SDC through the examination of species richness, which allowed us to incorporate all species into the analysis. We observed increased anuran species richness farther downstream from dams. Species least influenced by downstream distance from dams included two toad species (*Anaxyrus fowleri* and *A*.* americanus*) along with *L*. *catesbeianus*,* Gastrophryne carolinensis*, and *Hyla chrysoscelis,* species that may be considered less reliant on a natural flow regime and the variety of floodplain wetlands it supports. These two toad species are very terrestrial compared to the rest of our anuran assemblage and can use more permanent waterbodies for reproduction (Lannoo, 2005; Table 1). Similarly, while *H. chrysoscelis* and *G. carolinensis* generally use more ephemeral waterbodies for reproduction (Table 1), they will often breed in marginal habitats such as roadside ditches and retention ponds (Dorcas & Gibbons, 2008) or at the edges of permanent lentic habitats (Lannoo, 2005). *Lithobates catesbeianus* breeds in permanently inundated aquatic sites that are relatively unaffected by flow alteration (Fuller, Pope, Ashton, & Welsh, 2011), which may explain why their response was not as striking as other anurans in our study. Conversely, several species (i.e., *Acris crepitans*,* Anaxyrus terrestris*,* H. cinerea*,* Pseudacris crucifer*,* P*.* feriarum*,* L*.* clamitans*,* L. palustris*,* L. sphenocephalus*) were relatively sensitive to increasing distance downstream of dams, and these species tend to prefer ephemeral, relatively shallow breeding sites that hold enough water to host emergent aquatic vegetation but exclude fish predators (Butterfield, Lannoo, & Nanjappa, 2005; Gray, Brown, & Blackburn, 2005; Lannoo, 2005; Moriarty & Lannoo, 2005; Table 1). These specific requirements are less likely to occur in riparian zones that have reduced flooding frequency, particularly if the floodplain does not experience a strong enough hydrological connection to the river to sustain ephemeral water bodies. However, moving farther downstream of dams might allow tributaries to begin accumulating, thereby increasing habitat available for ephemeral breeders.

Urbanization is a pervasive source of habitat degradation that threatens anuran species (Gibbs, Whiteleather, & Schueler, 2005; Guzy et al., 2012; Hamer & McDonnell, 2008; Knutson et al., 1999). In a review of 32 urban studies investigating 40% of North American anuran species, Scheffers and Paszkowski (2012) found that amphibians as a whole respond negatively to urbanization, although responses may differ by species (e.g., Guzy et al., 2012; Rubbo & Kiesecker, 2005). Because urban wetlands tend to have less surrounding forest and longer hydroperiods that support fish predators, anuran species richness and abundance is often reduced, with the exclusion of ephemeral forest breeders (Gagné & Fahrig, 2007; Rubbo & Kiesecker, 2005) or species requiring forested uplands (Pillsbury & Miller, 2008). Urban watersheds alter microhabitats and facilitate the spread of exotic species that change prey communities and potentially outcompete native anurans (Riley et al., 2005). Furthermore, the negative effects of urbanization can be exacerbated in high‐traffic locations (Bee & Swanson, 2006; Pellet, Guisan, & Perrin, 2004). However, anuran species associated with riparian zones can persist even in urbanized areas (Dorcas & Gibbons, 2008) if natural habitat buffers are present (Hamer & McDonnell, 2010; Price, Snodgrass, & Dorcas, 2014) and connectivity with terrestrial habitat is maintained (McCarthy & Lathrop, 2011). Our results are consistent with previous research (Scheffers & Paszkowski, 2012) and suggest that anuran occupancy decreases when there is more urbanization surrounding study sites; however, our estimated mean community response to urbanization parameter distribution also included nontrivial support for positive values (95% CI −3.67 to 1.09). Variable anuran occupancy responses may have diluted the community response to urbanization. In addition, the urbanization response might have been poorly estimated relative to the influence of dams because the anuran community has had less time to be affected by urbanization pressure (Grummer & Leaché, 2017). In our study, dams were constructed in the 1800s and early 1900s, whereas significant urbanization pressure has only existed in recent decades. Finally, many of our sites were located along a State Scenic River, and our most urbanized study site only contained 49.3% urban land use, so our findings may not apply in landscapes with greater urbanization.

### 4.1 Caveats and limitations

We observed a strong relationship between increasing distance downstream of dams and anuran species richness, perhaps driven by impairment of flood plain inundation by flow regulation. However, downstream distance is likely a proxy measurement correlated with various structural or hydrological changes that accumulate farther downstream of dams (e.g., tributary accumulation; Ward & Stanford, 1995b), and because we cannot provide insight into specific mechanisms, it is important for natural resource managers to apply our findings cautiously. For example, changes in water temperature and chemistry, sediment accumulation, and channel incision might occur along a gradient downstream of dams, driven in part by peak stream‐flow discharge, dam height, hydraulic residence time of impoundments, and type of dam operation (Collier, Webb, & Schmidt, 1996; Ligon, Dietrich, & Trush, 1995; Poff & Hart, 2002; Pringle, Freeman, & Freeman, 2000). Therefore, determining connectivity of a river and its floodplain wetlands would benefit from information on daily discharge volume for each dam, in combination with measurements of overbank flows, rainfall, and consideration of structural components such as river gradient, width, and floodplain area.

### 4.2 Management recommendations

Our study supports SDC predictions for a meandering river and expands the SDC to include the riparian landscape. Distance downstream from dams is an important factor influencing anuran species richness, a pattern previously documented in fish (Cumming, 2004), riparian vegetation (Merritt & Wohl, 2006), and invertebrates (Ellis & Jones, 2013). Sites at the farthest distances downstream of dams (~50 km) had an estimated ~3 more species than those just below dams, a finding that is important for understanding ecological relationships in regulated rivers. Managing flows to ensure that riparian zones are inundated during amphibian winter and summer breeding seasons would likely benefit riparian amphibian communities. Such management will also increase connectivity of the riparian zone to the river channel, resulting in increased habitat heterogeneity that will benefit both aquatic and semi‐aquatic animals. Furthermore, avoiding aseasonal releases, which can displace adults, larvae, and/or eggs, would also benefit riparian amphibian communities. This could be achieved by using pre‐dam seasonal discharge data to identify an average discharge rate for each season, matching the discharge from an undammed tributary within the focal watershed to discharge below dams, and most importantly, basing real‐time alterations to flow releases on current environmental conditions such as increasing flow releases during current rain events (Lind et al., 1996). In addition, future studies should seek to elucidate mechanisms driving the patterns we observed, including the interactions between dams and number/size of tributaries and flow variation, as these may be important drivers structuring anuran assemblages along regulated rivers.

## CONFLICT OF INTEREST

None declared.

## AUTHOR CONTRIBUTIONS

SP and EE conceived the ideas and designed methodology; JG, SP, and EE collected the data; JG and BH analyzed the data; JG and SP led the writing of the manuscript. All authors contributed critically to the drafts and gave final approval for publication.

## DATA ACCESSIBILITY

Anuran occupancy data and site and sampling covariates available from the Dryad Digital Repository https://doi.org/10.5061/dryad.9qg57.
